# 21‐Hydroxylase Deficient Congenital Adrenal Hyperplasia Due to Maternal Uniparental Isodisomy

**DOI:** 10.1155/crie/3596318

**Published:** 2026-06-05

**Authors:** Michelle L. Kluge, Lynn Schema, Katy Schroepfer, Emily K. Thoreson, Kyriakie Sarafoglou, Ross A. Rowsey, Cherisse A. Marcou, Ann M. Moyer, Matthew J. Schultz

**Affiliations:** ^1^ Department of Laboratory Medicine and Pathology, Mayo Clinic, Rochester, Minnesota, USA, mayo.edu; ^2^ Department of Pediatrics, M Health Fairview University of Minnesota Masonic Children’s Hospital, Minneapolis, Minnesota, USA; ^3^ Public Health Laboratory, Minnesota Department of Health, St. Paul, Minnesota, USA, health.state.mn.us

**Keywords:** 21-hydroxylase deficient congenital adrenal hyperplasia 21-OHD CAH, CYP21A2, intrauterine growth restriction IUGR, uniparental disomy UPD

## Abstract

Congenital adrenal hyperplasia (CAH) due to 21‐hydroxylase deficiency (21‐OHD CAH) is an autosomal recessive genetic condition that results from pathogenic variants in the *CYP21A2* gene. Noncarrier parents are found in a small percentage of cases, typically due to de novo variants. However, uniparental disomy (UPD) should also be considered. UPD is generally suspected when an individual with an autosomal recessive condition is found to be homozygous for a rare pathogenic variant and consanguinity has been ruled out. However, UPD in *CYP21A2* may be hard to recognize because homozygous genotypes are not uncommon. We present the case of a 19‐month‐old male born preterm who presented to pediatric endocrinology after a positive newborn screen for CAH. He was small for gestational age, with a history of adrenal crisis and elevated 17‐hydroxyprogesterone (17‐OHP). Genetic testing revealed homozygosity for two common pathogenic *CYP21A2* variants: c.293‐13C >G and c.1360C >T (p.Pro454Ser), both variants were found in the heterozygous state in the mother while the father’s *CYP21A2* molecular testing was negative. UPD testing was pursued and the results were consistent with the patient having maternal isodisomy of chromosome 6. In cases of patients with CAH due to homozygous *CYP21A2* variants, suspicion for UPD may be increased if the child also presents with intrauterine growth restriction (IUGR), or transient neonatal diabetes (TNDM) mellitus, associated with maternal and paternal UPD of chromosome 6, respectively.

## 1. Introduction

Classic congenital adrenal hyperplasia (CAH) has a worldwide incidence of one in 14,000–18,000 and refers to a group of autosomal recessive inherited disorders that affect steroid biosynthesis [1]. Approximately 90%–95% of CAH cases result from 21‐hydroxylase deficiency (21‐OHD), which is caused by pathogenic variants in the *CYP21A2* gene [1, 2]. There is a spectrum of symptoms related to 21‐OHD CAH that are typically determined by the amount of 21‐hydroxylase residual enzyme activity. Broadly, patients have either classic or nonclassic CAH, and classic CAH is further subdivided into simple virilizing CAH (SV CAH) and salt‐wasting CAH (SW CAH). Patients with SW CAH typically have <1% residual enzyme activity and are at risk for life‐threatening SW crises if untreated. Generally, when a patient is found to have 21‐OHD CAH, both parents are carriers of heterozygous variants, and their offspring are compound heterozygous or homozygous for the familial pathogenic variants. However, in a small number of cases (~1%–2%), one parent is found to not be a carrier of the *CYP21A2* variants detected in their child [1]. Assuming correctly reported parentage, this result can be due to a de novo event (less likely with a rare and homozygous variant) or chromosomal uniparental disomy (UPD).

UPD, specifically uniparental isodisomy (UPiD, two identical copies of a chromosome inherited from the same parent), has been reported in several cases of patients with CAH and a noncarrier parent [3–9]. UPD is best known for disorders associated with imprinted regions such as Silver–Russell syndrome, Beckwith–Wiedemann syndrome, Prader–Willi syndrome, and Angelman syndrome [10]. However, UPiD can also unmask autosomal recessive variants and result in an affected child with an autosomal recessive condition if the two copies of an identical chromosome harbor a pathogenic variant [10]. UPiD is most often suspected when an individual is found to be homozygous for a rare variant in an autosomal recessive condition and the variant is only present in a single parent. Recognizing suspected UPD can be difficult in conditions such as 21‐OHD CAH because homozygous genotypes are common in this condition. Approximately 70%–75% of cases have one or more of nine common pathogenic pseudogene‐derived variants [1, 11]. These common variants result from gene conversion events between *CYP21A2* and its highly homologous pseudogene, *CYP21A1P*, which lies 30 kb upstream. We present a rare instance of 21‐OHD CAH due to maternal UPD, and highlight key signs that may increase a clinician or laboratorian’s suspicion of UPD in a case of 21‐OHD CAH.

## 2. Case Presentation

A newborn male presented to endocrinology with a presumed diagnosis of CAH. His prenatal history was notable for intrauterine growth restriction (IUGR) at 20 weeks and small kidneys. He was delivered at 36w4d after his mother presented with high blood pressure, headaches, and decreased fetal movement. At birth, the patient weighed 1720 g (3 lb 12.7 oz, 1st percentile) and his length was 42 cm (16.54″; 1st percentile). Head circumference was measured in the normal range at 33 cm (12.99″; 36th percentile). Normal male genitalia were noted at birth.

After delivery the patient was admitted to the NICU. The patient then had an adrenal crisis characterized by mottled appearance with poor perfusion and abnormal EKG–widened QRS complexes and peaked T waves. His electrolytes were significant for marked hyponatremia and hyperkalemia (sodium 124 mmol/L and K > 9 mmol/L). The same day, his newborn screen for CAH returned positive with 17‐hydroxyprogesterone (17‐OHP) >500 ng/mL (reference range: less than 15.1 ng/mL). A serum 17‐OHP was done and was reported as greater than 50,000 ng/dL. He was started on hydrocortisone, fludrocortisone, and sodium chloride at that time. The patient was discharged from the NICU after a 1‐month admission.

A diagnosis of SW CAH was suspected. Family history was negative for CAH and no reported family members had abnormal NBS.

Molecular genetic testing for 21‐OHD CAH was ordered (Mayo Clinic Laboratories, Rochester, MN). This test includes long‐range PCR with nested PCR amplicons sequenced by Sanger sequencing of the gene combined with multiplexed ligation‐dependent probe amplification copy number analysis to determine the genotype and discriminate between *CYP21A2* and its pseudogene *CYP21A1P*.

Copy number analysis for the patient detected two copies of *CYP21A2* and two copies of its highly homologous pseudogene *CYP21A1P*. Sequencing of *CYP21A2* revealed homozygosity for the common, pseudogene‐derived pathogenic variants c.293‐13C>G and c.1360C>T (p.Pro454Ser). The combined impact of c.293‐13C>G, a common SW variant with <1% enzyme activity, and p.Pro454Ser, a nonclassic variant with 20%–50% residual enzyme activity, is expected to result in a protein with no residual enzyme activity (a null allele). Homozygosity for this null allele is consistent with the patient’s diagnosis of SW CAH. However, sequencing was also notable for complete homozygosity of not only the two identified pathogenic variants but also five additional benign variants (Table 1).

**Table 1:** Sanger sequencing results for *CYP21A2* for the paternal, proband, and maternal samples.

Paternal	Proband	Maternal
	**c.-4C >T**	**c.-4C >T**
c.118C >T (p.Leu40 = )	**c.118C >T (p.Leu40 = )**	**c.118C >T (p.Leu40 = )**
c.138C >A (p.Pro46 = )	**c.138C >A (p.Pro46 = )**	**c.138C >A (p.Pro46 = )**
c.292+9C >T	**c.292+9C >T**	**c.292+9C >T**
c.293‐13C >A	** *c.293-13C >G* **	*c* *.293-13C >G*
c.318G >C (p.Pro106 = )		
		c.447 + 38C >T
c.550‐15C >A		
c.550‐8T >C		
c.739‐21C >T		
c.747C >G (p.Leu249 = )		
	** *c.1360C >T* (*p.Pro454Ser*)**	*c.1360C >T* (*p.Pro454Ser*)
	**c. ^∗^52C >T**	**c. ^∗^** **52C >T**

*Note:* c.DNA and protein nomenclature generated using transcript NM_000500.7 and build GRCh37/hg19. Bold font indicates homozygosity, pathogenic variants are italicized and underlined. If the font is not bold, italicized or underlined it indicates a benign heterozygous variant.

To further investigate the patient’s complete homozygosity of *CYP21A2* and to perform risk assessment for future pregnancies, parental samples were submitted for additional testing. Consanguinity and nonpaternity were denied. The patient’s father had two copies of *CYP21A2* and two copies of *CYP21A1P*. Sequencing of *CYP21A2* did not detect the c.293‐13C >G or p.Pro454Ser variant. However, sequencing revealed nine heterozygous benign variants, two of which were identified in the homozygous state in his son (Table 1). The patient’s mother was found to have two copies of *CYP21A2* and one copy of *CYP21A1P*. Sequencing of *CYP21A2* detected heterozygous c.293‐13C >G and p.Pro454Ser variants, as well as four homozygous benign variants and one heterozygous benign variant, all of which were also detected in her son. One additional heterozygous benign variant that was absent from her son was also identified (Table 1).

**Figure 1:**
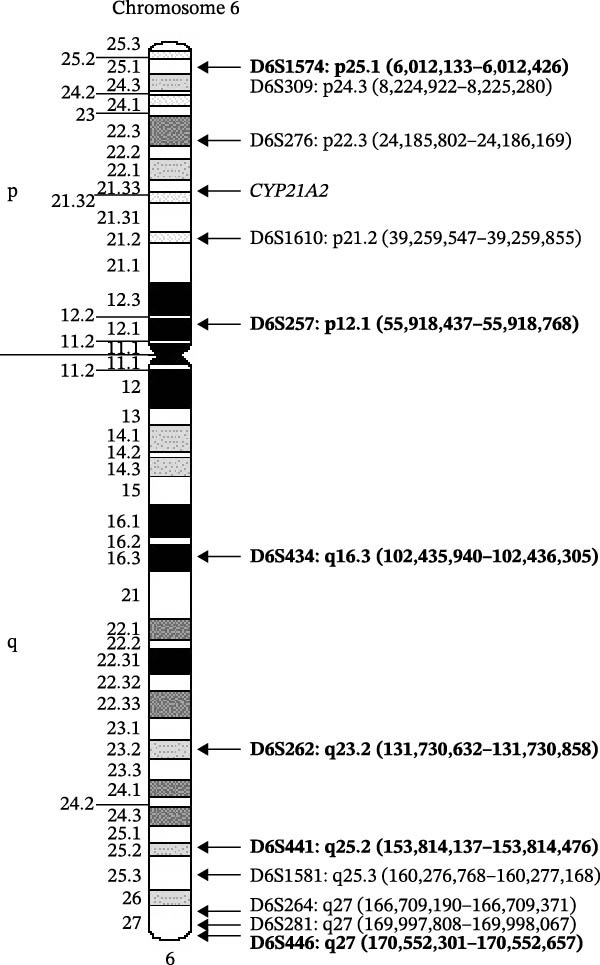
Ideogram of chromosome 6 with microsatellite markers used for UPD testing. Informative markers are shown in bold. *CYP21A2* gene locus is located at 6p21.33. Coordinates are provided using build GRCh37/hg19.

**Figure 2:**
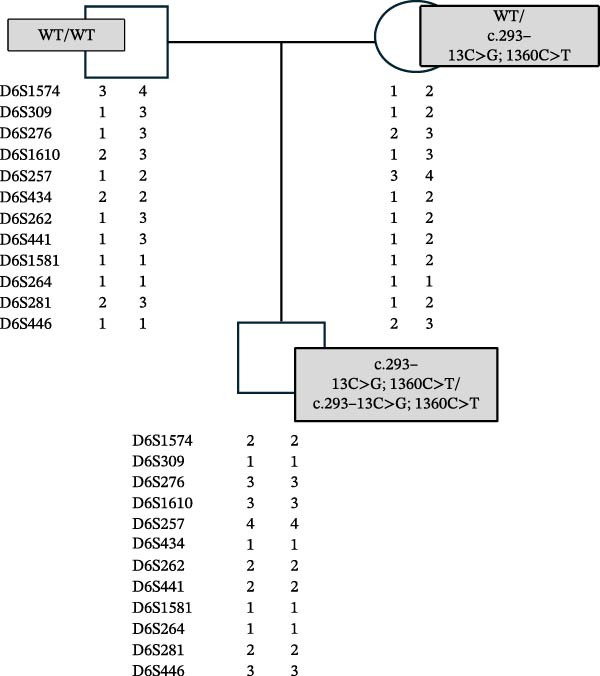
Family pedigree from UPD testing documenting the paternal, maternal and proband alleles. *CYP21A2* sequencing results are noted in gray boxes. Results are consistent with maternal isodisomy of chromosome 6.

Given the results, maternal UPiD was suspected. UPD testing (Mayo Clinic Laboratories, Rochester, MN) was ordered and six informative markers were identified (Figure 1). The chromosomal location of *CYP21A2*, 6p21.33, sits between two uninformative markers; however, given the culmination of data, this result was most consistent with maternal isodisomy of chromosome 6 (Figures 1 and 2).

The patient and his parents followed up with both genetics and pediatric endocrinology following the confirmation of his diagnosis of 21‐OHD CAH, classic, and SW type due to maternal UPiD. Following the confirmation of maternal UPiD, the recurrence risk for the patient’s parents to have another child with 21‐OHD CAH dropped from one in four (25%) to <1%. In addition, the recurrence risk for the proband’s extended paternal family members was reduced to population risk.

Early developmental milestones were on track and a neuropsychology evaluation at 19‐months of age using the Bayley‐4 Scales of Infant and Toddler Development revealed broadly average neurodevelopmental functioning. Throughout infancy the patient continued to have slow growth. At 1 year of age, he underwent a growth hormone stimulation test showing growth hormone resistance. He was subsequently started on growth hormone. While the patient continues to grow and gain weight, he remains below the 1st percentile for height and weight. Head circumference was most recently measured at the 1st percentile.

Due to his continued poor growth and concern for dysmorphic features, the patient was evaluated by a medical geneticist at age 19 months. Dysmorphic features including triangular appearing facies and cupped and anteverted ears were noted. A chromosomal microarray (CGH and SNP) as well as genetic testing for Silver–Russell syndrome were pursued. Genetic testing for Silver–Russell syndrome was negative. The CGH portion of the microarray was also normal; however, the SNP array identified UPD chromosome 1 as well as the already noted UPD of chromosome 6. UPD of chromosome 1 is not associated with any known phenotype; however, there remained the concern that it could result in homozygosity for a pathogenic variant, similar to this patient’s diagnosis of 21‐OHD CAH. Due to this concern, whole exome sequencing (WES) was ordered. WES results were consistent with maternal UPD of chromosomes 1 and 6 and confirmed previous findings of homozygous *HFE* c.845G >A, p.Cys282Tyr, confirming a diagnosis of autosomal recessive HFE‐associated hereditary hemochromatosis. Analysis of *CYP21A2* was not possible by WES methodology; however, the evidence was supportive of previously identified variants. A complex and maternally inherited variant of uncertain significance was detected in *NIPBL*, and a homozygous, maternally inherited likely pathogenic low penetrance allele was detected in *ABCA4*. However, neither of these variants were thought to contribute to the patient’s growth differences; therefore, no additional variants associated with the patient’s clinical presentation were detected by WES.

In order to estimate the number of cases where UPiD may be a possibility we retrospectively reviewed a year’s worth of cases tested in our laboratory for 21‐OHD CAH. We identified at least 26 patients who were homozygous for a pathogenic variant out of 264 cases. Due to testing including full gene sequencing of *CYP21A2* we were able to exclude one case based on a heterozygous benign variant; however, approximately 10% (25/264) of cases were homozygous for a pathogenic variant and all benign variants detected in the *CYP21A2* gene. The majority of these individuals (20/25) were homozygous for p.Val282Leu, which is the most common non‐classic CAH variant. Per the Genome Aggregate Database (gnomAD v.4.1.0), this variant has a general population prevalence of up to 1.8% in Admixed American individuals and 4.8% in Ashkenazi Jewish individuals [12]. There were an additional five cases (5/264, ~2%) which were completely homozygous for either full gene deletions or other pathogenic variants, where UPiD cannot be ruled out. Complete homozygosity in these cases could be explained by both parents being carriers for a common haplotype (as seen in p.Val282Leu, unpublished data) consanguinity, coincidence, or possible UPiD. This analysis excluded patients who were completely homozygous for benign variants (negative *CYP21A2* molecular testing result), which could also be an indicator of UPiD of chromosome 6 but not clinically impactful for 21‐OHD CAH.

## 3. Discussion

We present a case of SW 21‐OHD CAH due to maternal UPiD resulting in the patient being homozygous for the two *CYP21A2* pathogenic variants c.293‐13C>G and p.Pro454Ser. The patient’s mother was found to be a carrier of these two variants (variants are proven to be in cis, within the same copy of *CYP21A2*), while his father was found to not be a carrier of 21‐OHD CAH.

Literature on CAH, UPD of chromosome 6, as well as generic UPD review articles were reviewed. A total of four cases of 21‐OHD CAH due to UPD were identified, as well as one case of 3β‐HSD2 deficient CAH and one case of 11β‐hydroxylase deficient CAH [3–9].

The first case of 21‐OHD CAH due to UPD was reported by López‐Gutiérrez et al. [3] in a cohort of 21‐OHD patients from Mexico. The patient was female with ambiguous genitalia, hirsutism, hyperpigmentation, and moderate hyponatremia 4 days after birth. The reported patient was homozygous for c.293‐13C>G and p.Val282Leu. Both variants were detected in her father in the heterozygous state and her mother’s genetic testing was negative. Microsatellite analysis was consistent with paternal UPD of the short arm of chromosome 6 (6p) but indicated that UPD did not extend to the long arm of chromosome 6 (6q). The lack of 6q UPD was consistent with the fact that the reported patient did not have transient neonatal diabetes (TNDM), which is associated with an imprinted gene in the region of 6q22–q23 [3, 10].

The second case of 21‐OHD CAH due to UPD was reported by Spiro et al. [4]. The reported individual presented at 2.65 years of age with pubarche of 3 months duration, clitoral enlargement, and advanced bone age. The reported patient was born with IUGR, but her growth caught up to the 5^th^ percentile by 18 months and was at the 85^th^ percentile by her presentation at 2.65 years. The patient’s history was also notable for early motor delays. The patient was subsequently diagnosed with SV CAH and genetic testing revealed homozygous p.Ile173Asn, a common SV pathogenic variant, in *CYP21A2*. The reported patient’s mother was found to be heterozygous for this variant, and her father’s genetic testing was negative. Microsatellite analysis was consistent with maternal UPD based on seven informative markers [4].

The third reported case of 21‐OHD due to UPD was originally reported by Parker et al. [5], and then, referenced again by Finkielstain et al. [6]. This was the first reported male patient with 21‐OHD CAH due to UPD. The patient presented with IUGR, progressive respiratory distress due to persistent pulmonary hypertension, large patent ductus arteriosus, a positive NBS for CAH, and a clinical 17‐OHP level of 12,180 ng/dL (normal <200 ng/dL). A karyotype revealed the patient was mosaic for a marker chromosome (48,XXY, +mar [30]/47, XXY[20]), and microsatellite analysis revealed maternal UPiD of chromosome 6 and chromosome X. The reported patient was homozygous for a deletion of the *CYP21A2* gene. His mother was found to be compound heterozygous for p.Val282Leu and a deletion of the *CYP21A2* gene, indicating previously undiagnosed NC 21‐OHD CAH in the reported patient’s mother [5, 6].

The final case was reported in Eggermann et al. [9] as part of a larger review on maternal UPD of chromosome 6 (upd(6)mat). The reported male patient was delivered at 30w6d due to oligohydramnios and IUGR. He was noted to have adrenogenital syndrome, facial dysmorphia, clinodactyly of the 5^th^ digits, and flat valgus feet. He was diagnosed with SW 21‐OHD CAH due to a homozygous *CYP21A2* deletion. Follow‐up testing due to his persistent growth restriction indicated a normal male karyotype (46,XY) and SNP array confirmed large regions with isodisomy for chromosome 6, although he was noted to not be isodisomic for the *CUL7* locus (6p21.1). Parental *CYP21A2* testing results were not reported [9].

To compare our case to the previous four 21‐OHD UPD cases: all three cases of maternal UPD had IUGR [4, 5, 9]. Maternal UPD of chromosome 6 is rare, with about 20 cases being reported as of 2025; however, the majority of cases were notable for IUGR and preterm labor [13]. Our case further supports the association of maternal UPD of chromosome 6 with IUGR. Of note, our patient is still young, so it is unknown if his growth will normalize in a similar time frame to the patient presented by Spiro et al. [4] or if he will continue to exhibit persisting growth restriction as seen in Eggermann et al. [9].

All reported cases of 21‐OHD CAH due to UPD have had classic CAH. CAH is typically diagnosed clinically with a work‐up that includes serum 17‐OHP level, this work‐up may be follow‐up to a positive newborn screen, or family history, and genetic testing is not always performed. If a genetic diagnosis is pursued, parental testing is not always completed, or is done through carrier screening panels, which typically only include select *CYP21A2* variants due to the complex nature of this locus. This may mean that the true prevalence of UPD as a cause of 21‐OHD CAH is underestimated and underreported. In a retrospective review of a year of genetic testing in our laboratory we cannot exclude UPiD in approximately 10% of cases on the sole basis of an individual’s result.

This case report adds to the sparse literature about UPiD as a cause of 21‐OHD CAH and highlights the difficulty identifying patients with potential UPD due to the majority of pathogenic *CYP21A2* variants being common pseudogene‐derived variants. We suggest considering parental studies in cases where an affected individual exhibits homozygosity for all variants and UPiD cannot be excluded to refine recurrence risk for potential future pregnancies. Furthermore, our case highlights patient features that may raise suspicion of UPD in 21‐OHD CAH and demonstrates additional clinical utility of full gene *CYP21A2* testing compared with genotyping, the former of which can suggest complete *CYP21A2* gene homozygosity and potential UPiD.

## Author Contributions

All authors made individual contributions to authorship. Michelle L. Kluge, Emily K. Thoreson, Ann M. Moyer, Matthew J. Schultz, Cherisse A. Marcou, and Ross A. Rowsey were involved in diagnosis and molecular laboratory result interpretation and reporting. Katy Schroepfer, Lynn Schema, and Kyriakie Sarafoglou were involved in the diagnosis and management of the patient. Michelle L. Kluge and Matthew J. Schultz were involved in manuscript submission.

## Funding

No public or commercial funding was received.

## Disclosure

All authors have read and approved the final version of the manuscript. Matthew J. Schultz had full access to all of the data in this study and takes complete responsibility for the integrity of the data and the accuracy of the data analysis.

## Conflicts of Interest

The authors declare no conflicts of interest.

## Data Availability

Some or all datasets generated during and/or analyzed during the current study are not publicly available but are available from the corresponding author upon reasonable request.
